# Association of sleep duration and quality with elevated hs-CRP among healthy Korean adults

**DOI:** 10.1371/journal.pone.0238053

**Published:** 2020-08-25

**Authors:** Hwi-Won Lee, Hyung-Suk Yoon, Jae Jeong Yang, Minkyo Song, Jong-koo Lee, Sang-Ah Lee, Ji-Yeob Choi, Daehee Kang

**Affiliations:** 1 Department of Preventive Medicine, Seoul National University College of Medicine, Seoul, Korea; 2 Department of Biomedical Sciences, Seoul National University Graduate School, Seoul, Korea; 3 Department of Family Medicine, Seoul National University Hospital, Seoul, Korea; 4 Department of Preventive Medicine, Kangwon National University School of Medicine, Gangwon, Korea; University of Colorado Denver School of Medicine, UNITED STATES

## Abstract

This study aimed to investigate the association of sleep duration and quality with high-sensitivity C-reactive protein (hs-CRP) among middle-aged and elderly Koreans. Among a total of 74,867 participants (25,069 men and 49,798 women) recruited for the Health Examinees (HEXA) study, adjusted geometric means of hs-CRP level were compared across categories of sleep duration (<6, 6–7, 8–9, and ≥10 hours) and sleep quality (difficulty in initiating sleep and maintaining sleep) using ANCOVA models. Odds ratios (ORs) and 95% confidence intervals (CIs) for elevated hs-CRP (>3 mg/L) associated with sleep characteristics were estimated using multivariable-adjusted logistic regression models. Men who slept ≥10 hours per day were significantly associated with elevated hs-CRP (OR = 1.47, 95% CI 1.11–1.95). Whereas in women, difficulty in initiating sleep (OR = 1.28, 95% CI 1.04–1.57 for “Always”), and maintaining sleep was significantly associated with elevated hs-CRP levels (OR = 1.13, 95% CI 1.02–1.26 for “Often”; OR = 1.11, 95% CI 0.97–1.28 for “Always”). Additionally, women who experienced poor sleep quality presented an elevated level of hs-CRP (OR = 1.13, 95% CI 1.03–1.23). Our findings suggest that excessive sleep duration and poor sleep quality are significantly associated with the elevated inflammatory marker, specifically hs-CRP. Further research is needed to examine the effect of sleep interventions focused on these factors.

## Introduction

Sleep plays a crucial role in balancing the physiological functions of humans through a stable 24-hour cycle, otherwise known as the circadian rhythm. Studies have indicated that sleep loss and disturbances can induce alterations in the inflammatory, immune, metabolic, and neuroendocrine systems [1]. Furthermore, sleep-related imbalance of the circadian rhythm appears to correlate with an increased risk of cardiovascular diseases [2] and mortality [3].

Although the exact mechanism underlying sleep and adverse health outcomes remains elusive, emerging evidence suggests that sleep may intimately involve in human cardio-metabolic health by controlling the inflammatory processes [4]. Previous studies have shown a significant association of sleep deprivation with elevated inflammatory cytokines such as interleukin 6 (IL-6) and tumor necrosis factor α (TNF-α) and C-reactive protein (CRP), an acute-phase reactant [5–8]. Similarly, excessive sleep duration has also been associated with elevated inflammatory cytokines [7, 9]. Furthermore, other studies have indicated that both self-reported sleep complaints and poor sleep quality were linked with inflammatory state [10–13]; however, those associations were likely to be modified by sex. Among the various inflammatory biomarkers that indicate inflammation responses, CRP is one of the most widely used indicators that predicts the risk of cardiovascular diseases among the general population [14]. In particular, a highly sensitive assay of CRP (hs-CRP) that provides a more accurate detection rate at the microvascular level is currently used to evaluate the risk of future cardiac morbidity [15, 16]. Moreover, the level of hs-CRP is associated with individual health status and various factors, including acculturative stress [17], socioeconomic status [18], and depressive symptoms [19]. Previous studies investigated the effects of sleep disorders such as obstructive sleep apnea [20, 21] and insomnia [22] on hs-CRP levels. Several studies have evaluated the association between sleep characteristics and hs-CRP level among the general population [6, 9, 13, 23]; the results, however, derive from mainly western population or are conservative in sample size [24–28]. A 2009 study carried out by the Organization for Economic Cooperation and Development reported that Koreans ranked at the bottom of 18 countries for sleep time; a nationwide study found that nearly 20% of Korean adults were affected by insomnia, reflecting an increase from 2002 survey [29, 30]. The evident increase in negative sleep patterns among Korean adults calls for attention in the realm of social and public health, as potential hazards related to poor sleep hygiene may be more pronounced in this group of Asians.

In this study, we evaluated the sex-specific association of sleep duration and quality with hs-CRP level, and further explored the combined effects of sleep duration and quality on the increase in hs-CRP level. Using data from the Health Examinees (HEXA) study, we examined the putative association between sleep characteristics and inflammatory responses.

## Materials and methods

### Study population

The source of the present study is the large-scale genomic cohort, the HEXA study, in Korea. Detailed information on the HEXA study has been described elsewhere [31]. Briefly, participants for baseline study were recruited during two phases from health examination centers across the country: 2004–2008 and 2009–2012. All study participants provided written informed consent prior to entering the study. Information was collected through means of interview-based questionnaire, physical examinations as well as laboratory analyses of collected biological specimens. All participants are being followed up according to a standardized study protocol. The HEXA study undergoes an annual review by the Ethics Committee of the Korean Health and Genomic Study of the Korea National Institute of Health and the institutional review boards of all participating hospitals (IRB No. E-1503-103-657).

In this study, we analyzed participants from the first phase (2004–2008) of the HEXA study due to changes in the logistics regarding the measurement of hs-CRP. Among the 85,323 participants aged 40 to 69 years, respondents who failed to provide information on sleep duration and sleep quality (N = 2,639) were excluded. Those excluded from the analysis were omitted strictly based on missing information on the sleep-related variables thus there is low probability of non-random exclusion. Along with participants without information on hs-CRP (N = 79), individuals with hs-CRP >10 mg/L (N = 1,419) were excluded given the possibility of the presence of other clinically relevant inflammatory conditions. To minimize potential influence due to preclinical conditions and/or inflammatory changes, participants who reported as currently receiving treatment for chronic diseases (N = 6,319), specifically acute liver disease (N = 71), arthritis (N = 3,076), asthma / chronic bronchitis (N = 445), bladder infection (N = 245), cancer (N = 530), cholelithiasis (N = 94), chronic liver disease (N = 491), fatty liver disease (N = 491), gastritis (N = 1404), and tuberculosis (N = 50), were excluded from the analysis. This approach allowed us to acquire conservative results regarding the association between sleep characteristics and hs-CRP level. After these exclusions, a total of 74,867 participants consisting of 25,069 men and 49,798 women remained as the final analytic sample.

### Assessment of sleep duration and sleep quality

Self-reported sleep duration and sleep quality data was collected by in-person interview. Sleep duration was asked by the question, “On average, how many hours of sleep did you get per day during the past year (including nap time)?” Responses were categorized into <6 hours (short sleepers), 6–7 hours (average sleepers, reference group), 8–9 hours (moderately long sleepers), and ≥10 hours (excessive sleepers) [32]. The reference group for sleep duration is based on the results from the representative population-based sample of middle-aged Korean adults which consistently indicated that average sleep duration of Koreans was less than 7 hours per day (range 6.3–6.9 hours)[33]. Sleep quality was assessed through responses to two independent questions related to how participants felt both physically and psychologically in relation to two of the most common symptoms of insomnia: 1) difficulty in initiating or falling asleep and 2) difficulty in maintaining sleep. Difficulty in initiating sleep was addressed by the question, “Do you have difficulty falling asleep?” Answers were categorized as “not at all,” “sometimes,” “often,” and “always.” Difficulty in maintaining sleep was assessed by the question, “Do you struggle to get back to sleep when you wake up in the middle of the night?” Response categories were identical to those used in the previous question. To estimate participants’ overall sleep status, a composite score was created by summing the scores of difficulty in initiating sleep and maintaining sleep. Each response category (not at all, sometimes, often, and always) was given a numerical score from 0 to 3 in the order of increasing disturbances in sleep quality. The combination of difficulty in initiating sleep and maintaining sleep, therefore, had a potential score range of 0 to 6 and were subsequently classified into two groups as the following: score ranges 0–2 reflected good sleep quality, and score ranges 3–6 reflected poor sleep quality.

### Measurements of hs-CRP levels

Venous blood samples were drawn after a minimum of 8 hours overnight fasting in order to perform clinical chemistry tests. Blood samples were immediately separated by centrifugation and then sent to a central laboratory authenticated via external quality assessment. Levels of hs-CRP were quantified by particle-enhanced immunonephelometry with a detection threshold of 0.01 mg/L using Pureauto S CRP latex (Tokyo, Japan) on a Hitachi 7180. Values under the minimum detectable level were recorded as 0.01 mg/L. hs-CRP levels were categorized into two groups (≤3 mg/L and >3 mg/L) in which 3mg/L has been suggested to reflect the cutoff level for low and intermediate cardiovascular risk [16].

### Covariates

Our prior study on sleep has revealed significant association between sleep duration and various factors [32]. Covariates were selected *a priori* and included as follows: age at enrollment (numerical variable); educational attainment (middle school graduate or lower, high school diploma, college degree or higher); marital status (married or single/separated); and occupational status (non-manual worker, manual laborer, and economically inactive). Smoking status was categorized into two groups: non-current smokers (including both non-smokers (<400 cigarettes in lifetime) and ex-smokers) and current smokers (≥400 cigarettes during their lifetime and still smoking). Alcohol consumption was classified in the same manner (non-current or current drinkers), while physical activity divided participants into those who engaged in regular sweat-inducing exercise or those who did not engage in regular exercise. Highly trained medical staff were responsible for all anthropometric measurements. Height and body weight were assessed using a body composition analyzer with participants wearing only light clothing and no shoes. Body mass index was calculated as weight divided by height squared in meters (kg/m^2^). In accordance with the standard protocol, blood pressure measurements were taken twice with a 30-second interval from the right arm by using a standard mercury sphygmomanometer on participants who were seated comfortably. Both systolic and diastolic blood pressure readings were recorded and the mean values of the readings were calculated. Regarding the laboratory analyses, plasma level of fasting glucose, triglycerides, and high-density lipoprotein cholesterol were measured using standard enzymatic methods on overnight fasting blood samples.

### Statistical analyses

All statistical analyses were conducted separately by sex to investigate the gender-specific association between hs-CRP and sleep characteristics including sleep duration, difficulty in initiating sleep, and maintaining sleep. Given that the distribution of hs-CRP levels was skewed, hs-CRP values were log transformed, and the results were presented as raw and adjusted geometric means with 95% confidence intervals (CIs). Using logistic regression models, the odds ratios (ORs) and 95% CIs were estimated to evaluate the association between increased hs-CRP (>3 mg/L) and sleep characteristics while controlling for age only in the age-adjusted model. In the fully adjusted model, the following variables were included: age, education level, marital status, occupational status, smoking, alcohol drinking, physical activity, body mass index, lipid profiles, and current treatment status for hypertension and diabetes. To investigate the combined effect of sleep duration and sleep quality on hs-CRP levels, adjusted ORs with 95% CIs were also estimated for all combinations of sleep duration and sleep quality. All statistical analyses were performed using SAS software version 9.4 (SAS Institute, Cary, NC, USA).

## Results

Selected characteristics of the 74,867 participants are shown in Table 1, where men and women showed statistically significant differences. The mean age of the participants was 53.7 years and 52.0 years for men and women, respectively. Men were observed to have higher proportions of current smokers, current drinkers, and regular exercisers. Approximately 65% of men and 61% of women reported to sleep an average of 6–7 hours. Short (<6 h) and excessive (≥10 h) sleepers were more common among women (12.5% and 2.0%, respectively), and women appeared to experience more disturbances in sleep quality such as difficulty in initiating sleep (“Always” = 2.8% for women vs. 1.7% for men) and maintaining sleep (“Always” = 8.4% for women vs. 5.5% for men). The geometric means of hs-CRP across sleep duration and sleep quality are shown in S1 Table. Geometric mean levels of hs-CRP were higher in men compared to women, and excessive sleepers presented significantly higher levels of hs-CRP regardless of sex. Those who responded “Always” in difficulty in initiating sleep were independently associated with the increased hs-CRP level (Geometric mean = 0.96, 95% CI 0.92–0.99 for men; Geometric mean = 0.88, 95% CI 0.86–0.89 for women). Difficulty in maintaining sleep also revealed that respondents of “Always” reported high level of hs-CRP (Geometric mean = 0.95, 95% CI 0.93–0.97 for men; Geometric mean = 0.85, 95% CI 0.84–0.86 for women) (S1 Table).

**Table 1:** Basic characteristics of study participants.

	Men (N = 25,069)	Women (N = 49,798)
***Sociodemographic factors***		
Age group (years)		
40–49	8,694 (34.7)	20,731 (41.6)
50–59	9,332 (37.2)	19,603 (39.4)
60–69	7,043 (28.1)	9,464 (19.0)
Education level		
≤ Middle school	6,442 (25.7)	20,480 (41.1)
High school diploma	10,114 (40.3)	20,584 (41.3)
≥ College degree	8,083 (32.2)	7,862 (15.8)
Occupational status		
Non-manual	7,724 (30.8)	5,299 (10.6)
Manual	11,960 (47.7)	12,573 (25.3)
Unemployed	4,771 (19.0)	30,525 (61.3)
Marital status		
Married	23,685 (94.5)	42,643 (85.6)
Single or separated	1,278 (5.1)	6,918 (13.9)
Menopausal status		
No		20,744 (41.7)
Yes		27,187 (54.6)
***Lifestyle factors***		
Smoking status	17,110 (68.3)	48,329 (97.1)
Non-current smokers	7,869 (31.4)	1,078 (2.2)
Current smokers		
Alcohol drinking		
Non-current drinkers	6,974 (27.8)	34,359 (69.0)
Current drinkers	18,052 (72.0)	15,176 (30.5)
Physical activity		
Regular exercisers	13,924 (55.5)	25,177 (50.6)
Non-exercisers	11,106 (44.3)	24,532 (49.3)
***Sleep characteristics***		
Sleep duration per day (hours)		
< 6	2,569 (10.3)	6,228 (12.5)
6–7	16,294 (65.0)	30,218 (60.7)
8–9	5,742 (22.9)	12,373 (24.9)
≥ 10	464 (1.9)	979 (2.0)
Difficulty in initiating sleep		
Not at all	15,999 (63.8)	27,503 (55.2)
Sometimes	7,497 (29.9)	17,601 (35.3)
Often	1,155 (4.6)	3,327 (6.7)
Always	418 (1.7)	1,367 (2.8)
Difficulty in maintaining sleep		
Not at all	10,238 (40.8)	16,967 (34.1)
Sometimes	9,906 (39.5)	19,713 (39.6)
Often	3,541 (14.1)	8,966 (18.0)
Always	1,384 (5.5)	4,152 (8.3)
Sleep quality^a^		
Good	21,185 (84.5)	38,821 (78.0)
Poor	3,884 (15.5)	10,977 (22.0)

Values presented as n (%);

^***a***^Sleep quality was scored according to the sum of difficulty in initiating sleep and maintaining sleep; not at all, sometimes, most of time, and always were given a numerical score of 0 to 3 in the order of increasing order in sleep disturbance for each question. The total score range of 0 to 6 was divided into two categories: good sleep quality (0, 1, 2 points) and poor sleep quality (3, 4, 5, 6 points).

The association of sleep characteristics with hs-CRP level >3 mg/L is shown in Table 2. Men who reported to be sleeping ≥10 hours were associated with elevated hs-CRP level in the fully adjusted model (OR = 1.47, 95% CI 1.11–1.95), although sleep quality variables were not associated with elevated hs-CRP level among men. In women, long sleep duration was associated with elevated hs-CRP level in the fully adjusted model, but was marginally significant (OR = 1.26, 95% CI 0.98–1.62). Women who experienced difficulty in initiating sleep showed association with high level of hs-CRP (OR = 1.28, 95% CI 1.04–1.57 for “Always”), while marginally significant association was observed for difficulty in maintaining sleep (OR = 1.11, 95% CI 0.97–1.28 for “Always”). Poor sleep quality among women was significantly associated with hs-CRP >3 mg/L (OR = 1.13, 95% CI 1.03–1.23), but not men (OR = 1.01, 95% CI 0.90–1.15) (Table 2). In a stratified analysis according to hs-CRP level, among women who had an elevated level of hs-CRP (>3mg/L), short sleepers (<6 hours) were observed to experience poor sleep quality (OR = 1.30, 95% CI 1.06–1.60; S1 Fig).

**Table 2:** Odds ratios of sleep characteristics in association with hs-CRP level >3 mg/L by sex.

	Men (N = 25,069)				Women (N = 49,798)			
	≤3 mg/L	>3 mg/L	OR (95% CI)^a^	OR (95% CI)^b^	≤3 mg/L	>3 mg/L	OR (95% CI)^a^	OR (95% CI)^b^
	(N = 22,995)	(N = 2,074)			(N = 46,900	(N = 2,898)		
**Sleep duration per day (hours)**								
< 6	2,365 (10.3)	204 (9.8)	0.95 (0.82–1.11)	0.95 (0.81–1.10)	5,810 (12.4)	418 (14.4)	1.06 (0.95–1.19)	1.05 (0.94–1.18)
6–7	14,990 (65.2)	1,304 (62.9)	1.00 (Ref.)	1.00 (Ref.)	28,497 (60.8)	1,721 (59.4)	1.00 (Ref.)	1.00 (Ref.)
8–9	5,234 (22.8)	508 (24.5)	1.09 (0.98–1.22)	1.08 (0.97–1.20)	11,683 (24.9)	690 (23.8)	1.01 (0.92–1.10)	1.00 (0.91–1.09)
≥ 10	406 (1.8)	58 (2.8)	**1.52 (1.15–2.02)**	**1.47 (1.11–1.95)**	910 (1.9)	69 (2.4)	**1.29 (1.00–1.66)**	1.26 (0.98–1.62)
**Difficulty in initiating sleep**								
Not at all	14,661 (63.8)	1,338 (64.5)	1.00 (Ref.)	1.00 (Ref.)	25,966 (55.4)	1,537 (53.0)	1.00 (Ref.)	1.00 (Ref.)
Sometimes	6,893 (30.0)	604 (29.1)	1.00 (0.90–1.11)	0.99 (0.90–1.10)	1,6566 (35.3)	1,035 (35.7)	1.07 (0.99–1.16)	1.06 (0.98–1.15)
Often	1,062 (4.6)	93 (4.5)	0.98 (0.78–1.22)	0.93 (0.74–1.15)	3,103 (6.6)	224 (7.7)	1.18 (1.02–1.36)	1.15 (1.00–1.33)
Always	379 (1.6)	39 (1.9)	1.13 (0.81–1.58)	1.07 (0.77–1.50)	1,265 (2.7)	102 (3.5)	**1.30 (1.06–1.60)**	**1.28 (1.04–1.57)**
**Difficulty in maintaining sleep**								
Not at all	9,358 (40.7)	880 (42.4)	1.00 (Ref.)	1.00 (Ref.)	15,952 (34.0)	1,015 (35.0)	1.00 (Ref.)	1.00 (Ref.)
Sometimes	9,141 (39.8)	765 (36.9)	0.93 (0.84–1.03)	0.92 (0.84–1.02)	18,676 (39.8)	1,037 (35.8)	0.95 (0.86–1.03)	0.94 (0.86–1.03)
Often	3,237 (14.1)	304 (14.7)	1.03 (0.90–1.18)	1.01 (0.88–1.15)	8,389 (17.9)	577 (19.9)	**1.15 (1.03–1.28)**	**1.13 (1.02–1.26)**
Always	1,259 (5.5)	125 (6.0)	1.05 (0.86–1.28)	1.01 (0.83–1.23)	3,883 (8.3)	269 (9.3)	1.13 (0.99–1.30)	1.11 (0.97–1.28)
**Sleep quality**^c^								
Good	19,441 (84.5)	1,744 (84.1)	1.00 (Ref.)	1.00 (Ref.)	36,627 (78.1)	2,194 (75.7)	1.00 (Ref.)	1.00 (Ref.)
Poor	3,554 (15.5)	330 (15.9)	1.04 (0.92–1.18)	1.01 (0.90–1.15)	10,273 (21.9)	704 (24.3)	**1.15 (1.05–1.25)**	**1.13 (1.03–1.23)**

hs-CRP, high-sensitivity C-reactive protein; values presented as n (%);

^a^Adjusted for age

^b^Adjusted for age, education level, marital status, occupational status, smoking, alcohol drinking, physical activity, BMI, triglyceride, HDL-cholesterol, and current treatment status for hypertension and diabetes mellitus.

^c^Sleep quality was scored according to the sum of difficulty in initiating sleep and maintaining sleep; not at all, sometimes, most of time, and always were given a numerical score of 0 to 3 in the order of increasing order in sleep disturbance for each question. The total score range of 0 to 6 was divided into two categories: good sleep quality (0, 1, 2 points) and poor sleep quality (3, 4, 5, 6 points).

*Bold values indicate statistical significance.

## Discussion

In the current study including 74,867 Koreans, excessive sleep duration (≥10 h/day) and poor sleep quality appear to be associated with deteriorative alterations in the inflammatory system. Male excessive sleepers exhibited significantly elevated hs-CRP levels even after adjusting for all putative covariates. Moreover, disturbances in sleep quality (i.e., difficulty in initiating sleep and maintaining sleep) were related to elevated hs-CRP in women.

Previous studies have explored the association of sleep duration with changes in circulating inflammatory profiles. Experimental research indicates that various immune and inflammatory parameters, including IL-6, TNF-α, hs-CRP, leukocytes, neutrophil counts, and natural killer cells are activated as a result of deprivation of sleep duration and/or deterioration of sleep quality [5, 8, 34–36]. Epidemiological studies also support the possibility that insufficient sleep may be linked to fluctuations in inflammatory molecules [6, 7, 10, 37]. Furthermore, a significant association between excess sleep and pro-inflammatory conditions has been reported [7, 9, 10]. Consistent with the previous findings, our findings indicate that length of sleep beyond the average sleep duration (6–7 h/day) may be associated with an elevated level of hs-CRP. This tendency was most pronounced among long sleepers. Men who slept for more than 10 hours per day showed an increased mean level of hs-CRP. On the other hand, the effect of short sleep duration on variations in hs-CRP level was not significantly distinct from the effect of an average sleep duration when potential covariates were fully adjusted. The biological mechanisms between long sleep durations and inflammatory conditions are not yet fully understood. Nonetheless, it has been speculated that several intermediates may play a role, including depressive symptoms, lower socioeconomic status, and pre-existing illnesses [38, 39]. Given that alterations in the nocturnal interval of circadian rhythms can induce systematic change involving neuroendocrine function and body temperature for example [40], there may exist possible biological switches that underlie inflammatory pathways and interact with excessive sleep duration. Poor quality of sleep appears to interfere with the immune and inflammatory systems.

While some inconsistencies have been found, the majority of previous studies indicate a positive correlation between poor sleep quality and inflammatory status [10–13]. Some of these inconsistencies may be explained by sex differences: sleep disturbances are more frequent among women, with the magnitude of sleep comorbidities also being more severe in women [9]. In contrast, a large population-based epidemiological study in Finland reported that moderate-to-severe sleep disturbances was associated with higher hs-CRP levels in men, but not in women [13].

Similarly, our findings showed that women who frequently reported poor sleep qualities, such as difficulty in initiating sleep or maintaining sleep, were significantly more likely to have elevated hs-CRP levels. It is likely that the stronger association in women is a result of gender differences in stress reactivity as it is generally reported that women are more vulnerable to stress-related conditions and are more likely to be at-risk for psychopathology [41]. Sleep disturbances in women may also act as a considerable stressor, which may suppress the immune function in addition to exacerbating the inflammatory conditions, because greater hormonal variation in women is related to menopausal status [42, 43]. Future studies will need to identify the involvement of precise biological processes in order to truly elucidate the pathways by which sleep disruption causes adverse health consequences through manipulation of the immune system.

In the present study, we investigated whether the combined effects of inappropriate sleep duration and poor sleep quality would be associated with variations of hs-CRP level. Although explicit joint association could not be determined, the combination of inappropriate sleep duration (<6 hours) and poor sleep quality was significantly associated with higher hs-CRP level (>3mg/L) among women. Considering that sleep deprivation has been identified as an independent stressor that exerts influence on the regulation of immune function [44, 45], sleep-related stress may aggravate inflammatory response and result in additional health harms.

Our findings are derived from one of the largest cohorts in Korea, allowing us to develop our understanding of the association between disruption in sleep parameters and inflammatory status among healthy adults in Asia. Our results not only provide insight into a specific Asian population, it offers cross-cultural information that can be used to compare sleep parameters among different groups of populations. In our study, blood was drawn after a minimum of 8-hour fasting, in the morning time. The consistency in blood sampling time assures that the circadian effects on hs-CRP may be steady throughout our study samples. Moreover, the putative covariates affecting both sleep characteristics and immunological responses were successfully accounted for in the multivariate models and diminished other residual confounding effects.

Despite the methodological strengths, we note several limitations in our current study. First, as sleep duration and quality were assessed by self-reported information, we cannot rule out the possibility of potential bias and/or measurement errors. However, self-reported sleep duration is generally regarded to be strongly correlated with actigraphic monitoring results [46] and although ‘difficulty in initiating sleep’ and ‘difficulty in maintaining sleep’ was assessed using two simple questions, they are the most common symptoms of insomnia and thus relevant variables of evaluation. Moreover, sleep duration may vary according to time of the year, etc., we indicated in the questionnaire the average number of hours during the past year, which reflects the overall sleep duration. Second, a cross-sectional design did not allow us to firmly establish a causal relationship, thus our results require careful consideration with regard to directionality of the interpretation. Likewise, since hs-CRP levels were measured only once at the time of the baseline survey, intra-individual variations in hs-CRP levels due to individual health conditions or latent diseases could not be taken into account. Moreover, we cannot overlook the possibility that those who sleep >10 hours may have underlying conditions that may be independently related to inflammatory response. Further studies will need to embrace a prospective follow-up approach and utilize a repeated measures design in order to detect chronological changes and to properly investigate the long-term effects of sleep disruption on immunological perspectives. Third, factors regarding obstructive sleep apnea were not measured, so health effects of sleep quality such as snoring cannot be determined and absence of such factor may affect the results. Bearing such limitation in mind, we have thus designed the analysis to obtain conservative results. Previous research shows that elevated hs-CRP levels are seen in the US population who sleep for 4 hours or less [4, 9]. Our risk estimates on short sleep, on the contrary, may be somewhat attenuated since our questionnaire used pre-defined category for sleep duration responses, where short sleep was defined as 6 hours or less. Future analysis on short sleepers split into subgroups may help examine the effect of extreme sleep durations on inflammatory biomarkers. Lastly, this study was performed in Korea; thus the findings cannot be generalized to other nations or ethnic groups. A collaborative research encompassing diverse population would be an ideal future project in rendering a fuller picture regarding sleep duration and hs-CRP.

In conclusion, excessive sleep duration and poor sleep quality appear to be significantly associated with inflammatory response among healthy Korean adults. Further research is warranted to examine the effect of sleep and other lifestyle interventions focused on these factors.

## Supporting information

S1 Table Geometric mean of hs-CRP across sleep characteristics. Levels of hs-CRP depend on sleep duration and sleep quality.
(DOCX)

S1 Fig Stratified analysis according to hs-CRP level in women. In women with hs-CRP level >3mg/L, the odds ratios of poor sleep quality depend on the duration of sleep.
(DOCX)
