# Supplementary material for: Association of sleep duration and quality with elevated hs-CRP among healthy Korean adults
Source: PLoS One. 2020 Aug 25;15(8):e0238053. doi: 10.1371/journal.pone.0238053 (PMC7446961; doi:10.1371/journal.pone.0238053)
Supplement: S1 Table — Levels of hs-CRP depend on sleep duration and sleep quality. (DOCX) [file pone.0238053.s001.docx]

**S1 Table. Geometric mean of hs-CRP across sleep characteristics. Levels of hs-CRP depend on sleep duration and sleep quality.**

|  |  | **Men (N = 25,069)** | | |  | **Women (N = 49,798)** | | |
| --- | --- | --- | --- | --- | --- | --- | --- | --- |
|  |  | **N** | **Geometric mean** | **95% CI** |  | **N** | **Geometric mean** | **95% CI** |
| Sleep duration per day |  |  |  |  |  |  |  |  |
| < 6 hrs. |  | 2,569 (10.3) | 0.92 | 0.90-0.93 |  | 6,228 (12.5) | 0.86 | 0.85-0.87 |
| 6 - 7 hrs. |  | 16,294 (65.0) | 0.92 | 0.91-0.92 |  | 30,218 (60.7) | 0.83 | 0.82-0.83 |
| 8 - 9 hrs |  | 5,742 (22.9) | 0.93 | 0.93-0.94 |  | 12,373 (24.9) | 0.83 | 0.83-0.84 |
| ≥ 10 hrs. |  | 464 (1.9) | 0.98 | 0.94-1.01 |  | 979 (2.0) | 0.86 | 0.84-0.88 |
| Difficulty in initiating sleep |  |  |  |  |  |  |  |  |
| Not at all |  | 15,999 (63.8) | 0.92 | 0.92-0.93 |  | 27,503 (55.2) | 0.83 | 0.82-0.83 |
| Sometimes |  | 7,497 (29.9) | 0.92 | 0.91-0.93 |  | 17,601 (35.3) | 0.83 | 0.83-0.84 |
| Often |  | 1,155 (4.6) | 0.92 | 0.90-0.94 |  | 3,327 (6.7) | 0.85 | 0.84-0.86 |
| Always |  | 418 (1.7) | 0.96 | 0.92-0.99 |  | 1,367 (2.8) | 0.88 | 0.86-0.89 |
| Nonrestorative sleep |  |  |  |  |  |  |  |  |
| Not at all |  | 10,238 (40.8) | 0.92 | 0.91-0.93 |  | 16,967 (34.1) | 0.83 | 0.83-0.84 |
| Sometimes |  | 9,906 (39.5) | 0.92 | 0.91-0.92 |  | 19,713 (39.6) | 0.82 | 0.82-0.83 |
| Often |  | 3,541 (14.1) | 0.93 | 0.92-0.94 |  | 8,966 (18.0) | 0.84 | 0.84-0.85 |
| Always |  | 1,384 (5.5) | 0.95 | 0.93-0.97 |  | 4,152 (8.3) | 0.85 | 0.84-0.86 |
| Sleep complaints^a^ |  |  |  |  |  |  |  |  |
| Score-0 |  | 8,459 (33.7) | 0.92 | 0.91-0.93 |  | 12,882 (25.9) | 0.82 | 0.82-0.83 |
| Score-1 |  | 6,891 (27.5) | 0.92 | 0.91-0.93 |  | 13,026 (26.2) | 0.83 | 0.82-0.83 |
| Score-2 |  | 5,835 (23.3) | 0.92 | 0.92-0.93 |  | 12,913 (25.9) | 0.83 | 0.83-0.84 |
| Score-3 |  | 2,524 (10.1) | 0.93 | 0.92-0.95 |  | 6,506 (13.1) | 0.84 | 0.84-0.85 |
| Score-4 |  | 925 (3.7) | 0.94 | 0.92-0.96 |  | 2,933 (5.9) | 0.85 | 0.84-0.86 |
| Score-5 |  | 242 (1.0) | 0.92 | 0.87-0.96 |  | 873 (1.8) | 0.86 | 0.84-0.88 |
| Score-6 |  | 193 (0.8) | 0.94 | 0.90-0.99 |  | 665 (1.3) | 0.86 | 0.84-0.89 |
| Total score of sleep complaints^b^ |  |  |  |  |  |  |  |  |
| Lower sleep complaints |  | 21,185 | 0.92 | 0.91-0.92 |  | 38,821 | 0.83 | 0.82-0.83 |
| Higher sleep complaints |  | 3884 | 0.93 | 0.92-0.94 |  | 10,977 | 0.85 | 0.84-0.85 |

^a^Sleep complaints were scored according to the sum of difficulty in initiating sleep and nonrestorative sleep; not at all, sometimes, often, and always were given a numerical score of 0 to 3 in the order of increasing complaint for each question.

^b^The total score range of 0 to 6 was divided into lower sleep complaints (0, 1, 2 points) and higher sleep complaints (3, 4, 5, 6 points)
