# Supplementary material for: Association of sleep duration and quality with elevated hs-CRP among healthy Korean adults
Source: PLoS One. 2020 Aug 25;15(8):e0238053. doi: 10.1371/journal.pone.0238053 (PMC7446961; doi:10.1371/journal.pone.0238053)
Supplement: S1 Fig — In women with hs-CRP level >3mg/L, the odds ratios of poor sleep quality depend on the duration of sleep. (DOCX) [file pone.0238053.s002.docx]

**Figure S1. Stratified analysis according to hs-CRP level in women. In women with hs-CRP level >3mg/L, the odds ratios of poor sleep quality depend on the duration of sleep.**


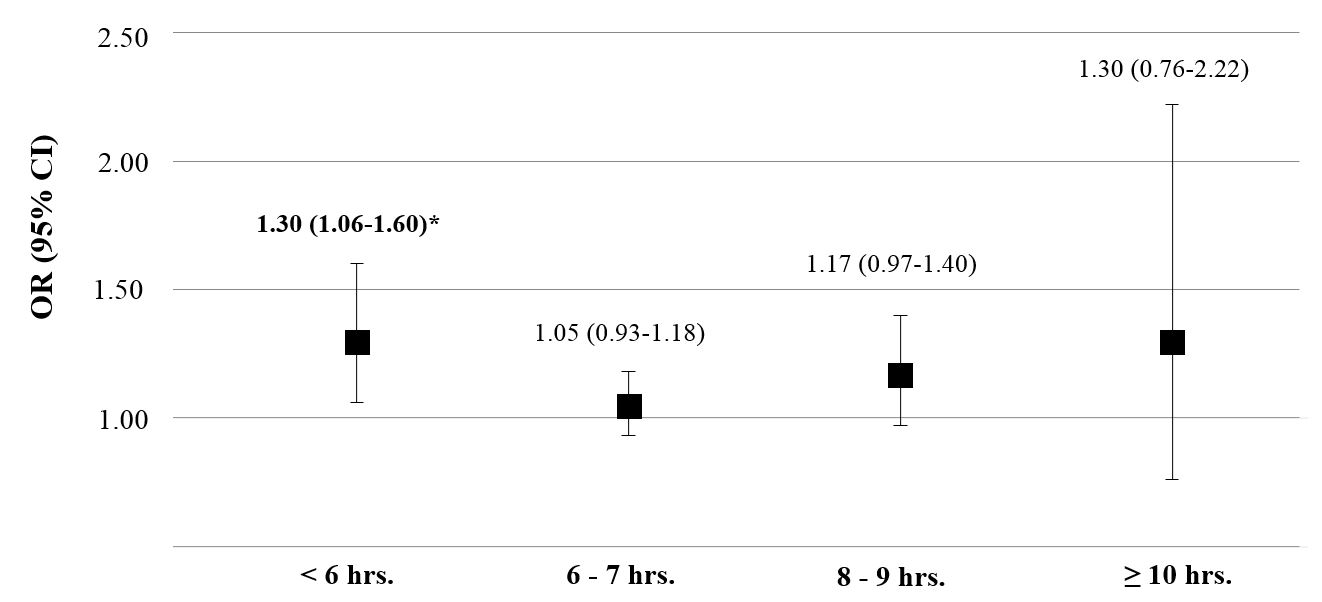


hs-CRP, high-sensitivity C-reactive protein; OR, odds ratio; CI, confidence interval. Odds ratios adjusted for age, education level, marital status, occupational status, smoking, alcohol drinking, physical activity, BMI, triglyceride, HDL-cholesterol, current treatment status for hypertension and diabetes mellitus. Sleep quality was scored according to the sum of difficulty in initiating sleep and nonrestorative sleep; not at all, sometimes, most of time, and always were given a numerical score of 0 to 3 in the order of increasing order in sleep disturbance for each question. The total score range of 0 to 6 was divided into two categories: good sleep quality (0, 1, 2 points) and poor sleep quality (3, 4, 5, 6 points).

^*^Bold value indicates statistical significance.
